# Artificial limb representation in amputees

**DOI:** 10.1093/brain/awy054

**Published:** 2018-03-09

**Authors:** Fiona M Z van den Heiligenberg, Tanya Orlov, Scott N Macdonald, Eugene P Duff, David Henderson Slater, Christian F Beckmann, Heidi Johansen-Berg, Jody C Culham, Tamar R Makin

**Affiliations:** 1 Institute of Cognitive Neuroscience, University College London, London, UK; 2 FMRIB Centre, Nuffield Department of Clinical Neuroscience, University of Oxford, Oxford, UK; 3 Donders Institute for Brain, Cognition and Behaviour, Radboud University Nijmegen, Nijmegen, The Netherlands; 4 Neurobiology Department, Life Sciences Institute, Hebrew University of Jerusalem, Jerusalem, Israel; 5 Brain and Mind Institute, Department of Psychology, University of Western Ontario, Canada; 6 Oxford Centre for Enablement, Oxford University Hospitals NHS Foundation Trust, Oxford, UK; 7 Wellcome Centre for Human Neuroimaging, University College London, London, UK

**Keywords:** plasticity, body representation, reorganization, neuroimaging, motor control

## Abstract

The human brain contains multiple hand-selective areas, in both the sensorimotor and visual systems. Could our brain repurpose neural resources, originally developed for supporting hand function, to represent and control artificial limbs? We studied individuals with congenital or acquired hand-loss (hereafter one-handers) using functional MRI. We show that the more one-handers use an artificial limb (prosthesis) in their everyday life, the stronger visual hand-selective areas in the lateral occipitotemporal cortex respond to prosthesis images. This was found even when one-handers were presented with images of active prostheses that share the functionality of the hand but not necessarily its visual features (e.g. a ‘hook’ prosthesis). Further, we show that daily prosthesis usage determines large-scale inter-network communication across hand-selective areas. This was demonstrated by increased resting state functional connectivity between visual and sensorimotor hand-selective areas, proportional to the intensiveness of everyday prosthesis usage. Further analysis revealed a 3-fold coupling between prosthesis activity, visuomotor connectivity and usage, suggesting a possible role for the motor system in shaping use-dependent representation in visual hand-selective areas, and/or vice versa. Moreover, able-bodied control participants who routinely observe prosthesis usage (albeit less intensively than the prosthesis users) showed significantly weaker associations between degree of prosthesis observation and visual cortex activity or connectivity. Together, our findings suggest that altered daily motor behaviour facilitates prosthesis-related visual processing and shapes communication across hand-selective areas. This neurophysiological substrate for prosthesis embodiment may inspire rehabilitation approaches to improve usage of existing substitutionary devices and aid implementation of future assistive and augmentative technologies.

## Teaser

Can the brain repurpose neural resources originally developed to support hand function for the control of artificial limbs? By studying individuals with congenital or acquired hand-loss using functional MRI, van den Heiligenberg *et al.* show that prosthesis usage shapes brain activity and connectivity. Neural resources can be repurposed to support artificial limbs.

## Introduction

Our hands are the primary tool of the brain, and the loss of a hand leads to profound changes in individuals’ abilities to interact with their environment (Makin *et al.*, 2013*a*; Hahamy *et al.*, 2017). Since brain organization is thought to be shaped by experience (Ejaz *et al.*, 2015), real-life constraints on behaviour, such as hand loss, should provide a powerful driver for brain reorganization (Pons *et al.*, 1991; Flor *et al.*, 2006; Makin *et al.*, 2015). It has recently been suggested that the profound reorganization observed following hand loss possibly occurs to accommodate changes in individuals’ abilities to interact with their environment in daily life (Makin *et al.*, 2013*a*; Hahamy *et al.*, 2015, 2017). Specifically, it has been suggested that the territory of the missing limb could be reappropriated to support the representation of other body parts that substitute the missing hand function as a compensatory strategy. A key strategy for adapting to hand loss is using an artificial limb (hereafter ‘prosthesis’). Prosthesis usage strongly depends on both motor control and visual information, particularly considering the limited somatosensory inputs from the artificial limb.

It is well established that within the primary somatosensory and motor cortex (SI/M1, respectively) specific areas show strong selectivity for inputs and outputs relating to the hand (Penfield and Rasmussen, 1950; Kaas *et al.*, 1979). It has been demonstrated that similar hand-selectivity also exists in the visual system. Specifically, areas in the lateral occipitotemporal cortex show visual selectivity for upper limbs compared to other body parts (Orlov *et al.*, 2010) or object categories (Bracci *et al.*, 2010, 2012). The functional relationship between hand representations in the sensorimotor and visual systems is still unknown (Tal *et al.*, 2016). In recent years, evidence has been accumulating to demonstrate that visual hand-selective areas are involved in action perception and cognition (Gallivan and Culham, 2015; Lingnau and Downing, 2015; Downing and Peelen, 2016). However, it is still unclear to what extent representation in this area is informed by personal daily-life motor experience.

Here we examined individuals with hand-loss to determine how alternative motor strategies through prosthesis usage affect functioning of—and coupling between—visual and sensorimotor hand-selective areas. Participants (hereafter ‘one-handers’; Table 1) with either unilateral acquired arm amputation (*n = *16) or unilateral congenital maldevelopment of the hand (*n = *16) with varying degrees of prosthesis usage underwent a functional MRI session. The functional MRI session comprised task-based scans in which the participants viewed images of different categories (e.g. upper limbs, prosthetic arms) and a resting state scan.

**Table 1:** Individuals’ demographic details and daily prosthesis usage habits

Subject	Gender	Age	Deprivation age	Level of amputation	Missing hand side	Cause	Usage frequency (MAL)	Usage time		
								Cosmetic	Mechanical	Myoelectric
PA01	M	57	20	Below elbow	Left	Trauma	0.57	5	0	0
PA02	F	49	0	Below elbow	Left	Congenital	0.46	4	0	0
PA03	M	59	40	Above elbow	Left	Trauma	0	0	0	0
PA04	F	52	0	Below elbow	Right	Congenital	0.15	5	1	0
PA05^a^	M	58	27	Above elbow	Left	Trauma	0.09	5	2	0
PA06	M	53	28	Below elbow	Left	Trauma	0.24	3	5	0
PA07	M	52	0	At wrist	Left	Congenital	0.04	0	3	0
PA08	M	41	27	Above elbow	Right	Trauma	0.09	2	1	0
PA09	M	48	17	Above elbow	Left	Trauma	0	2	2	0
PA10	F	25	0	At wrist	Right	Congenital	0	0	0	0
PA11	M	49	0	Above elbow	Left	Congenital	0.26	1	4	0
PA12	M	37	27	Above elbow	Left	Trauma	0.28	0	2	0
PA13	F	46	38	Below elbow	Left	Trauma	0	0	0	0
PA14	F	28	0	At wrist	Left	Congenital	0	0	0	0
PA15	M	64	33	Below elbow	Right	Trauma	0.33	0	2	5
PA16	M	38	0	Below elbow	Left	Congenital	0.39	5	0	0
PA17	F	24	18	Below elbow	Right	Trauma	0	0	0	0
PA18	F	27	0	Below elbow	Left	Congenital	0.54	5	0	0
PA19	M	49	37	Above elbow	Left	Trauma	0	1	0	0
PA20	M	60	0	At wrist	Left	Congenital	0.06	2	0	0
PA21	F	34	0	Below elbow	Right	Congenital	0.46	5	0	0
PA22	F	36	0	Below elbow	Right	Congenital	0.57	5	0	0
PA23	F	50	45	Above elbow	Left	Tumour	0	0	2	0
PA24	F	41	0	Below elbow	Left	Congenital	0.54	0	0	5
PA25	M	29	24	Through shoulder	Left	Trauma	0.09	0	0	2
PA27	M	25	0	Below elbow	Left	Congenital	0.59	1	0	5
PA28	M	34	0	At wrist	Left	Congenital	0.11	0	0	3
PA29	M	25	18	At wrist	Left	Trauma	0	0	2	0
PA30	M	38	0	Below elbow	Left	Congenital	0	0	2	1
PA31	F	49	0	At wrist	Left	Congenital	0	1	0	0
PA32	M	45	20	Below elbow	Right	Trauma	0.09	2	0	0
PA33	M	32	31	Above elbow	Left	Trauma	0	0	2	0

^a^This participant was excluded from the functional MRI analysis.

M/F = male/female; MAL = Motor Activity Log scores: how frequently one-handers use their prosthesis in an inventory of 27 daily activities (e.g. taking money out of wallet etc.). Scores of 0 = never, 1 = sometimes, 2 = very often. The sum of all items was divided by the highest possible score, such that individuals were rated on a scale ranging from 0 to 1. Prosthesis usage time relates to wear time, 1–5: the scale for prosthesis usage time: 0 = never; 1 = rarely; 2 = occasionally; 3 = daily, <4 h; 4 = daily, 4–8 h, 5 = daily, >8 h.

We hypothesized that reappropriation of hand-selective cortical resources depends on successful implementation of the prosthesis in daily life to substitute the missing hand’s function. Therefore, we predicted that more frequent habitual prosthesis usage (which strongly depends on visual feedback; Antfolk *et al.*, 2013) would increase processing for prostheses in typically hand-selective visual areas and increase cross-talk between visual and sensorimotor hand-selective areas. To investigate the role of visual experience further, we presented participants with images of both the prosthesis belonging to them (i.e. highly familiar), and images of a prosthesis exemplar belonging to another one-hander, unfamiliar to the observer. This allowed us to determine whether the representation of prosthetic limbs depends on specific experience, or rather more general categorical representation. Finally, we tested 24 able-bodied controls with varying degrees of passive visual exposure to prosthesis usage (Supplementary Table 1). We predicted that the degree to which individuals are passively exposed to prostheses usage should not scale with functioning of—and coupling between—visual and sensorimotor hand-selective areas.

Upper limb prosthetic limbs are broadly classified in two subcategories: active prostheses (affording adjustment of grip), and passive cosmetic prostheses. Active prostheses include: (i) mechanical prostheses, typically having little visual similarity to a hand and operated via the opposite shoulder to adjust grip size; and (ii) myoelectric prostheses, affording adjustment of grip based on ipsilateral arm-muscles while also having a visual appearance resembling a hand. Passive prostheses (hereafter ‘cosmetic’) are typically designed to resemble in visual appearance the human hand and arm but are not operational. Nevertheless, it is important to emphasize that passive prostheses can greatly enhance daily lives functionality, and are in fact preferred by the majority of amputees for daily functioning (Jang *et al.*, 2011; Østlie *et al.*, 2012, see also Table 1). We therefore tested our predictions considering both active and cosmetic prosthesis types.

## Materials and methods

### Participants

Thirty-two individuals with a missing hand [one-handers, mean age 42.3 years, standard deviation (SD) = 11.8, 12 females, eight missing their right hand] were recruited to take part in the study (Table 1). Sixteen one-handers lost their hand because of amputation (mean years since amputation: 16.7), and 16 had congenital unilateral hand absence (amelia). In addition, 24 age- and gender-matched two-handed controls (mean age 41.7 years, SD = 13.1; 12 female; eight left-handed) took part in the study (Supplementary Table 1). Recruitment was primarily carried out through Opcare (contracting prosthetic providers for National Health Services, UK) in accordance with Oxford University’s Medical Sciences inter-divisional research ethics committee (Ref: MSD-IDREC-C2-2014-003). Informed consent and consent to publish was obtained in accordance with ethical standards set out by the Declaration of Helsinki. One additional one-hander was recruited to the study but did not participate in the scanning session because of claustrophobia. Two control participants did not complete the motor functional MRI task because of time constraints. Another one-hander was excluded from data analysis because of poor quality of neuroimaging data.

### Experimental procedures

Participants took part in a single experimental session, involving questionnaires, behavioural tasks [as reported in Hahamy *et al.* (2017) and van den Heiligenberg *et al.* (2017); see osf.io/kd2yh for the full study protocol] and an MRI session. Questionnaires included demographic and clinical details (as summarized in Table 1), phantom sensations and pain (as described in Makin *et al.*, 2013*b*), and prosthesis usage (as described below).

### Experience with prosthetic limbs

#### Prosthesis usage habits

Prosthesis usage habits in one-handers are summarized in Table 1 and Supplementary Table 2. Of the 32 one-handers, two were not prosthesis owners and another three did not use their prosthesis currently. Of the remaining 27, 17 one-handers regularly used an active prosthesis, either body powered (mechanical; *n = *11) or powered via electrical muscle signal (myoelectric; *n = *4) or both (*n = *2). Seventeen one-handers regularly used a cosmetic prosthesis (of which seven were also active prosthesis users, whereas the other 10 used a cosmetic prosthesis exclusively).

#### Usage measurements in one-handers

Daily prosthesis usage was assessed using a revised version of the Motor Activity Log (MAL) as described and validated by Makin *et al.* (2013*a*) and Hahamy *et al.* (2017). In brief, participants rated how frequently they use their prosthesis in an inventory of 27 daily activities, requiring varying degrees of motor control (e.g. taking money out of wallet; zipping up a coat; peeling fruit skin etc.). As this inventory was not exhaustive, and it is possible that participants wear the prosthesis for other purposes than stated in the inventory (e.g. for cosmetic purposes), participants additionally rated how much time they typically spend wearing their prostheses in their daily lives. Individuals’ MAL scores and wear time for the different types of prostheses are detailed in Table 1. Participants primarily using cosmetic (*n = *14) and active (*n = *13) prostheses similarly use their prosthesis in daily tasks, as reflected in both MAL scores [average ± standard error of the mean (SEM) cosmetic = 0.25 ± 0.06; active = 0.19 ± 0.06; group difference *t*(25) = 0.67, *P = *0.51] and usage time (average ± SEM cosmetic = 3.5 ± 0.45; active = 3.23 ± 0.38; group difference Mann-Whitney U-test = 84.5, *P = *0.77).

Both the MAL and maximum wear-time ratings were standardized using a Z-transform and summed to create a usage score that included both wear time and incorporation of the prosthesis in day-to-day activities, as previously implemented (van den Heiligenberg *et al.*, 2017). Note that the two variables composing the usage score highly correlated with each other [*n = *32, *r*(30) = 0.84; *P < *0.001].

#### Passive visual exposure to prosthesis usage in control participants

Fourteen of the control participants were family members or friends of prosthesis-using one-handers, or had professional relationships with prosthesis users. We asked each control participant to rate how frequently they observed artificial limbs being used for daily purposes, using the same procedures as described above. This involved both prosthesis observation log (POL) of the inventory of daily activities included in the MAL, and prosthesis observation time (analogous to wear time). Visual experience was quantified using the same approach implemented for active prosthesis usage, as detailed above. Three of the control participants did not complete the questionnaires and were therefore discarded from this analysis. The remaining 21 control participants showed a diverse range of visual experience of prosthesis usage (Supplementary Table 1).

### Experimental design: functional MRI tasks

#### Visual task

Visual stimuli consisted of photographs from the following five categories (Fig. 2A and B): (I) upper limbs; (II) man-made objects; (III) participants’ ‘own’ prosthesis; (IV) unfamiliar cosmetic prostheses; and (V) unfamiliar active prostheses. Four other categories, not relevant for the purpose of the present study, were also presented during functional MRI scans but are not reported here (see osf.io/kd2yh for full details).

Images of (I) upper limbs (with and without the arm, from both first and third person perspectives); and (II) man-made objects, which are typically non-manipulable, were taken from an online database. To generate stimuli for the prosthesis conditions III–V, one-handers were asked to bring their prostheses to the study with them. Pictures of each participants’ prosthesis were taken by the experimenters prior to the functional MRI session from different angles (both first and third person perspectives). In the (III) ‘own’ prosthesis condition, all one-handers who had brought their prosthesis to the study were presented with images of their own prostheses, either cosmetic or active (*n = *26, see Supplementary Table 2). For individuals using several prostheses, we used the prosthesis worn more often. All other participants (i.e. the remaining six one-handers who did not bring a prosthesis and all control participants) were shown instead pictures of their own shoe. This was done to fill gaps in the experimental time course. Shoes were selected as a familiar external object that was intended to exert similar cognitive effects (e.g. in terms of arousal) as the prosthesis, and therefore minimize differences in the scan time course across groups. Consequently, the shoe condition was not included in further analysis.

Images of cosmetic and active prostheses (taken from the one-handers prostheses image pool) were shown to all the study participants in the remaining (IV) and (V) conditions, respectively. This time, however, participants with a prosthesis were presented with images of another person’s prosthesis (i.e. not their own). Note that the subset of myoelectric prosthesis users (Supplementary Table 2) were shown another myoelectric prosthesis. For both upper-limb and prosthesis images, the hand/prostheses were matched to the one-handers’ missing-hand side and the non-dominant hand in controls (e.g. participants missing their left hand were presented with ‘left-handed’ hands/prostheses).

Visual stimuli had their background removed, normalized for size, placed on an equi-luminant grey background and overlaid with a fixation point. The experiment included four separate runs. In each of the runs, each visual condition comprised nine trials (i.e. nine condition repetitions). In each such trial a single image was shown for 1.5 s, followed by 2.5 s of fixation. Eight different exemplars of a particular image category were used in these nine trials: seven images were presented only once while one image was shown twice in succession. Participants were required to detect these repetitions and report them with a button press (one-back recognition task). This design resulted in 36 repetitions of the same condition across all runs (nine trials × four runs). The run order was varied across participants. First-order counterbalancing of the image sequences was performed using Optseq (http://surfer.nmr.mgh.harvard.edu/optseq), which returns the most optimal image presentation schedule. The specifics of this design were validated against an event-related design with a jittered interstimulus interval and a block design during piloting (*n = *4). Visual stimuli were presented on a screen located at the rear end of the scanner bore and were viewed through a mirror mounted on the head coil. Stimulus presentation was controlled by a Macbook-Pro running the Psychophysics Toolbox in Matlab (The MathWorks, Natick, MA).

#### Motor localizer

To localize the sensorimotor hand-selective area, participants were visually instructed to move their intact hand (dominant hand in controls) or feet (bilateral toe movements). Other body-part conditions, not relevant for the purpose of the present study, were also included in the scan but not reported here (see Hahamy *et al.*, 2017 and osf.io/kd2yh for full details). The protocol consisted of alternating 12-s periods of movement and rest. Each of the conditions was repeated four times in a quasi-counterbalanced order. Participants received training before the scan on the degree and form of the movements prior to the scanning session.

#### Resting state scan

During the resting state functional MRI scan, participants were instructed to keep their eyes open, look at a central fixation cross displayed on the screen, and let their mind wander.

### Data acquisition

All data were acquired using a 3 T Verio scanner (Siemens) with a 32-channel head coil. Anatomical data were acquired using a T_1_-weighted magnetization prepared rapid acquisition gradient echo sequence (MPRAGE) with the parameters: repetition time = 2040 ms; echo time = 4.7 ms; flip angle = 8°, voxel size = 1 mm isotropic resolution. Blood oxygenation level-dependent (BOLD) functional MRI during the resting state and visual task was acquired using a multiband-6 sequence (Uğurbil *et al.*, 2013) with the parameters: voxel size = 2 mm isotropic, repetition time = 1300 ms; echo time = 40 ms; flip angle = 66°. Seventy-two slices with 2 mm thickness and no slice gap were acquired in the oblique axial plane, covering the whole cortex and most of the cerebellum. Two hundred and fifty-six volumes in each of the visual runs and 230 volumes in the resting state scan were acquired. Additional dummy volumes were acquired before the start of each scan to achieve equilibrium. The first dummy volume was saved and later used as a reference for co-registration.

During the motor task, BOLD functional MRI was acquired using a multiple gradient echo-planar T_2_*-weighted pulse sequence, with the parameters: voxel size = 3 mm isotropic, repetition time = 2000 ms; echo time = 30 ms; flip angle = 90°; imaging matrix = 64 × 64; field of view = 192 mm axial slices. Forty-six slices with slice thickness of 3 mm and no gap were acquired in the oblique axial plane, covering the whole cortex, with partial coverage of the cerebellum. Additional dummy volumes were acquired before the start of the scan.

### Neuroimaging data processing and low-level analysis

All imaging data were processed using FMRIB’s Expert Analysis Tool (FEAT; version 6.0) of FMRIB’s Software Library (FSL; version 5.0). Subsequent analyses were performed using Matlab (version 7.11, The Mathworks Inc, Natick, MA).

#### Preprocessing

The following preprocessing steps were applied to each participant’s task data: motion correction using FMRIB’s Linear Image Registration Tool (MCFLIRT; Jenkinson *et al.*, 2002), B0-unwarping, brain extraction using BET (Smith, 2002), high-pass temporal filtering of frequencies below a cycle of 100 s and 130 s for visual and resting scans, respectively, grand-mean intensity normalization of the entire functional run by a single multiplicative factor, and spatial smoothing using a Gaussian kernel with a full-width at half-maximum of 3 mm for visual and resting scans and 5 mm for motor scans. Functional images were aligned to structural images initially using FMRIB’s Linear Image Registration Tool (FLIRT; Jenkinson and Smith, 2001; Jenkinson *et al.*, 2002) and then optimized using Boundary-Based Registration (Greve and Fischl, 2009). Structural images were transformed into MNI space using FMRIB’s Nonlinear Image Registration Tool (FNIRT).

#### Individual level statistical analysis

Statistical analysis was performed using FMRIB’s Improved Linear Model (FILM). We applied a general linear model (GLM), as implemented in FEAT, to each functional run, as detailed below.

##### Task-based scans

Each of the experimental conditions was modelled separately against rest (fixation). Regressors were created by convolving stimulus presentation with a double-gamma haemodynamic response function (HRF). For each run, six head motion parameters were included as regressors of no interest. In case of large movement between volumes (>1 mm) additional regressors of no interest were included in the GLM to account for each of these instances individually. Contrasts for the conditions of interest were defined either against a control condition (objects, to control for interindividual variations in visual activity; or feet, to control for motor task demands which are not hand specific, for the visual and motor task, respectively) or against the baseline. For the visual task, second-level analyses were conducted on each participant's four experimental runs using a fixed-effects analysis.

##### Resting state scan

To account for non-neuronal noise that might bias functional connectivity analyses (Behzadi *et al.*, 2007; Whitfield-Gabrieli and Nieto-Castanon, 2012), we extracted the BOLD time series underlying white matter and CSF in the resting state scans. For this purpose, the T_1_-weighted structural scans were segmented into grey matter, white matter, and CSF, using the segmentation algorithm available in the SPM12 software package. The white matter maps were restricted by the white matter standard mask from the Harvard-Oxford atlas and thresholded to select 30 000 voxels with the highest intensity values. The resulting maps were eroded by one voxel in each direction to minimize partial voluming with grey matter. This yielded white matter maps that contained 16 105± 68 voxels. CSF maps were created by thresholding the individual CSF maps to select the 2000 voxels with the lowest intensity. No erosion was applied.

For white matter and CSF maps, the first five eigenvectors were calculated using the preprocessed resting state time series, as they best characterize the majority of observed signal variation across a set of voxels within a region (Behzadi *et al.*, 2007; Whitfield-Gabrieli and Nieto-Castanon, 2012). Additionally, we extracted time series representing head motion throughout the scan in six directions. The 16 regressors of no interest were regressed out from the preprocessed resting state time series. The resulting time series (residuals) were z-transformed and subsequently used in region of interest-based connectivity analyses (see below).

#### Regions of interest

Since the focus of the study was on hand-selective areas, our main analysis was restricted to the individualized regions of interest.

##### Visual regions of interest

**Figure 1:**
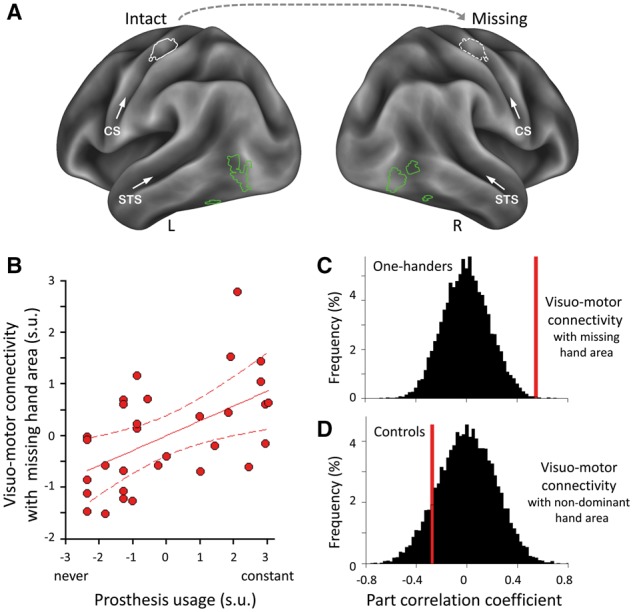
**Stronger connectivity between hand-selective areas in the visual and sensorimotor systems relates to greater prosthesis usage.** (**A**) Individualized regions of interest in an example participant. Hand-selective voxels were identified in lateral occipitotemporal cortex bilaterally by contrasting responses to hand versus object images (green) or in SI/M1 unilaterally by contrasting intact-hand (or dominant hand in controls) versus feet movements (white). The putative sensorimotor missing-hand area was estimated (hatched white) by mirror projecting the intact hand region of interest across hemispheres. CS = central sulcus; R/L = right/left hemispheres; STS = superior temporal sulcus. (**B**) Visuo-motor functional connectivity (between bilateral lateral occipitotemporal cortex and missing-hand SI/M1): correlations with prosthesis usage in one-handers. Visuomotor connectivity with the intact hand sensorimotor region of interest was regressed out of the missing hand visuo-motor measure. Scatter diagram is fitted with regression line and associated 95% confidence intervals; s.u. = standardized units. (**C** and **D**) Correlations for visuomotor connectivity with prosthesis usage (**C**) and observance (**D**) in one-handers and controls, respectively. Permutation tests of the null distributions (black) show that the correlation between visuo-motor connectivity and prosthesis usage (red) is significantly greater in one-handers than chance, but not in controls.

Bilateral visual hand-selective regions of interest were selected in lateral occipito-temporal cortex using the contrast hands versus objects (Fig. 1A). Note that visual hand representation is unchanged by hand loss (Striem-Amit *et al.*, 2017). For each participant, the 250 most hand-selective voxels were selected in each hemisphere (see Supplementary Fig. 1 for group probabilistic maps). Only voxels with a Z-score > 2 were included. Voxel selection was restricted to the superior temporal, middle temporal, inferior temporal, fusiform and parahippocampal gyri, as well as the lateral occipital cortex and occipitotemporal cortex (all bilateral), as defined by Harvard-Oxford atlas (Desikan *et al.*, 2006). Contrary to the motor hand representation, there is no clear laterality of visual hand representation in the occipitotemporal cortex (Shmuelof *et al.*, 2006). Voxels from both hemispheres were therefore combined in a single region of interest for use in subsequent analyses. We confirmed this by splitting the regions of interest across the hemispheres and performing the same analyses outlined below, in which we found no significant differences between the hemispheres.

##### Motor regions of interest

The putative sensorimotor missing-hand territory (or controls’ non-dominant hand territory) was identified in S1/M1 by mirror-projecting the intact hand region of interest (or controls’ dominant hand) across the mid-sagittal plane (Fig. 1A). To delineate the intact/dominant hand regions of interest, we used the contrast of intact or dominant hand (in one-handers or controls, respectively) versus feet movements. For each participant, the 200 most active voxels (with a Z-score > 2) were selected during intact/dominant hand movements in the contralateral sensorimotor cortex. For the two control participants who did not complete the motor functional MRI task, sensorimotor hand regions of interest were defined based on the corresponding group statistical maps, while taking into account participants’ hand-dominance. Voxel selection was restricted by the precentral and postcentral gyri, as defined by the Harvard-Oxford atlas.

To validate this flipping procedure, we repeated the analysis described in Hahamy *et al.* (2017). Specifically, we created a new region of interest for the non-dominant hand in each of our controls who completed the motor localizer scan (*n = *22). We generated this region of interest by selecting the 200 most active voxels in the contralateral sensorimotor cortex during non-dominant hand movements (versus feet movements), as described above. We then extracted the activation level, i.e. contrasts of parameter estimates (COPEs) for non-dominant hand movement under both the dominant-hand flipped region of interest and the non-dominant hand unflipped region of interest. The correlation coefficient across control participants between both regions of interest was high [*r*(20) = 0.82, *P < *0.001] suggesting that the flipped region of interest captured most of the interindividual variance of the relevant representation.

#### Region of interest analysis

##### Region of interest analysis of visual experiment

**Figure 2:**
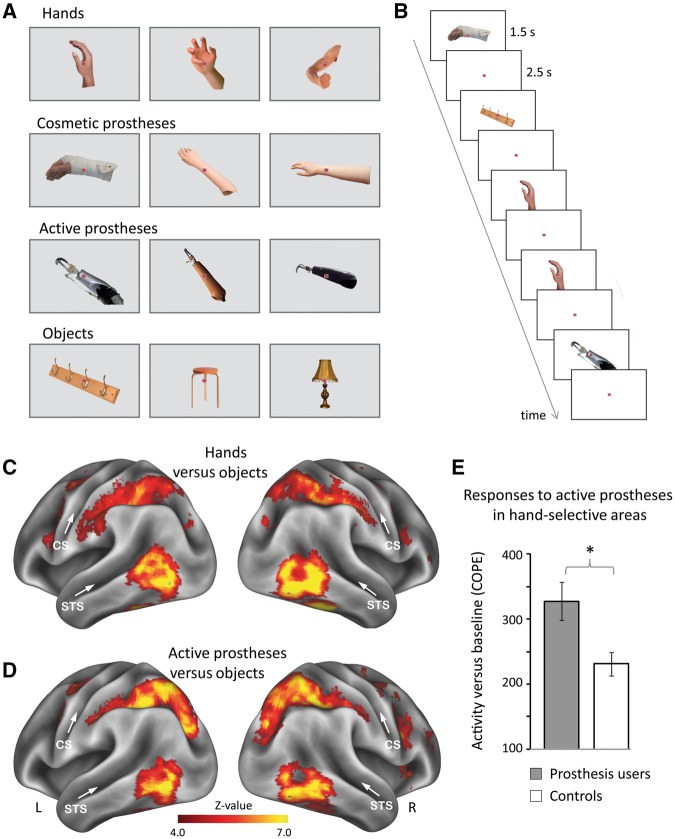
**Experimental design and brain activity.** (**A**) Example stimuli of hands, cosmetic and active prostheses, and objects. Participants’ own prostheses were included (‘own’ condition) as well as prostheses from other participants (‘other’s’ condition). (**B**) Stimuli were presented in an event-related design, involving a one-back recognition task. (**C** and **D**) Whole-brain activity maps for (**C**) hands and (**D**) active prostheses versus objects across all participants. Prostheses images activated lateral occipito-temporal cortex, partially overlapping with hand-selective activity. CS = central sulcus; R/L = right/left hemispheres; STS = superior temporal sulcus. (**E**) Prosthesis users (*n = *26) show stronger activity than controls in response to active prostheses in the visual hand area. COPE = contrasts of parameter estimates. Error bars indicate SEM. *P = 0.008.

The mean COPEs across voxels were extracted for each condition of interest (own/cosmetic/active prostheses) and control condition (objects) versus baseline. For the analysis of unfamiliar (others’) prostheses shown in Fig. 2E, we only included one-handers who regularly use cosmetic or active prostheses (*n = *26, see Supplementary Table 2 for full details). To account for differences in non-category-specific visual activity, as well as other sources of variance across participants of no interest, we regressed out object activity from prostheses activity (Van Breukelen, 2006). We performed a semi-partial (hereafter ‘part’) Pearson correlation between activity values for each condition of interest and prosthesis usage (or visual exposure to prosthesis usage in controls). For the unfamiliar (others’) conditions we included all amputees eligible for analysis (*n = *31; Supplementary Table 2), including the few individuals who do not use a prosthesis, allowing us to best assess usage-related variance. For the ‘own’ condition we could only include the participants who brought their own prosthesis to the study (*n = *25, Supplementary Table 2). Note that object activity didn’t correlate with prosthesis usage [*r*(23) = −0.11 and r(29) = 0.04 for ‘own’ and ‘unfamiliar’ conditions, respectively].

##### Region of interest analysis of resting state data

The purpose of this analysis was to determine intrinsic functional connectivity between visual hand selective areas and the missing-hand sensorimotor cortex. We focused on the missing hand sensorimotor cortex because the prosthesis is designed to substitute the missing hand’s motor function. However, given the known shared resting state variance across the two hand areas (Biswal *et al.*, 1995), we needed to account for any visuomotor connectivity that is shared between the two sensorimotor hand areas. For this purpose, the mean time series were extracted from the sensorimotor intact/dominant and missing/non-dominant hand regions of interest independently (in one-handers/controls, respectively). Next, we calculated the Pearson’s bivariate correlation between the time series of each of the two sensorimotor hand regions of interest and the mean time series of bilateral visual hand regions of interest. The correlation coefficients, after applying a Fisher’s Z-transform, represented the coupling between sensorimotor and visual areas in each participant. To assess the relationship between prosthesis usage (or visual exposure to prosthesis usage) and functional connectivity, we calculated the part correlation between prosthesis usage and missing hand sensorimotor-to-visual connectivity, while accounting for intact-hand sensorimotor-to-visual connectivity. This was done to ensure that the visuomotor connectivity we are measuring expresses the unique contribution of the missing hand sensorimotor cortex. For this purpose, we first adjusted the visuomotor connectivity strength between the S1/M1 missing hand region of interest with the bilateral visual region of interest, based on visuomotor connectivity with the intact hand region of interest. We then correlated the residual values of missing hand visuomotor connectivity with prosthesis usage. Note that visuomotor connectivity with the intact hand region of interest associated negatively (although not significantly) with prosthesis usage [*r*(29) = −0.18]. This suggests that visuomotor connectivity described below may also be influenced by prosthesis disuse (resulting in increased reliance on the intact hand, and increased visuomotor connectivity with the intact-hand region of interest).

### Statistical analysis

**Figure 3:**
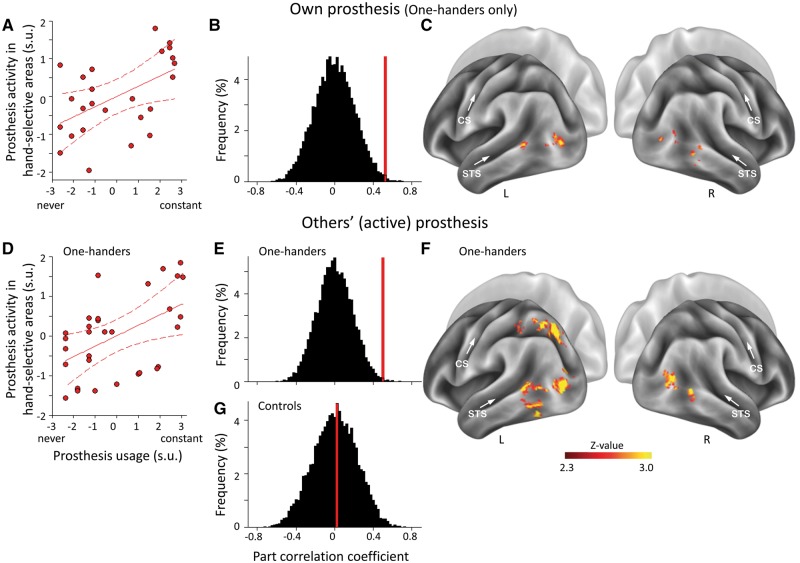
**Activity for prostheses in visual hand-selective areas relates to prosthesis usage.** Correlations between prosthesis usage and prosthesis activity in lateral occipito-temporal cortex for one’s own prosthesis (**A–C**) and exemplars of unfamiliar (others’) active prostheses (**D–F**). Correlations within individuals’ visual hand-selective regions of interest (**A** and **D**) and whole-brain analysis (**C** and **F**) are presented. Correlation was calculated while controlling for objects activity to achieve prosthesis-specific variations in functional MRI signal. Correlations of prosthesis-related activity with prosthesis usage are significantly greater in one-handers than chance (**B** and **E**), but not with passive observation in controls (**G**). CS = central sulcus; R/L = right/left hemispheres; STS = superior temporal sulcus.

Statistical analysis was calculated using SPSS (version 16.0, SPSS Inc., Chicago, IL) and Matlab (version 7.11, The Mathworks Inc, Natick, MA). After verifying normality (Shapiro-Wilks test), we used ANOVA and two-tailed Student’s *t*-tests to compare between groups and subgroups (or Mann-Whitney U-test, if the assumption of normality was violated). To determine whether the distribution of passive observation and prosthesis usage is overlapping, an independent-samples Kolmogorov-Smirnov test was applied. To examine relationship between brain measurements and prosthesis usage we ran permutation tests. To generate a chance distribution, the usage/visual experience scores were permuted 10 000 times, and a part Pearson correlation was repeated. We then assessed the significance of the true correlation coefficient, by calculating the two-sided *P*-values in the generated chance distributions (Figs 1C, D and 3B, E and G). To compare between two correlation coefficients, we used the Fisher’s r to Z test.

To examine joint versus unique relations between prosthesis usage, prosthesis activity and visuomotor connectivity, we performed a hierarchical linear regression analysis—a comparison of nested regression models. The dependent variable was visual activity to (unfamiliar) active prostheses (after accounting for object-related activity using part correlation, as described above). We used this active prosthesis condition because the active prosthesis least resembles a natural hand. Independent variables were prosthesis usage and resting state functional connectivity between the sensorimotor missing hand region of interest and the bilateral visual regions of interest (after accounting for intact hand visuomotor connectivity, as described above). A series of linear regression analyses was performed. By comparing the explained variance of a model containing both usage and connectivity (‘full model’) with regression models containing these variables as separate predictors (‘reduced models’), we assessed the unique and shared variance explained by these predictors.

Finally, to determine whether the correlations with prosthesis usage were affected by (i) primary prosthesis type for daily usage (active, cosmetic); and (ii) developmental period of hand-loss (congenital, adulthood), the main correlation analyses were repeated as above but with subgroup affiliation as a covariate. For this purpose, we carried out a one-way analysis of covariance (ANCOVA) on activity/connectivity values (after applying part correlation to account for objects activity/intact hand connectivity, as described above), with prosthesis usage as a main factor, and subgroup as a covariate, in separate analyses.

#### Group level analysis of task-based data

To further confirm and extend our main region of interest results and to determine whether the reported effects were specific to lateral occipitotemporal cortex, we also carried out whole-brain analyses (note, however, that such analysis is not appropriate for regions showing strong lateralization with respect to the amputation side). Whole-brain activity maps across all participants [for hands versus objects and unfamiliar (other’s) active prostheses versus objects, Fig. 2C and D] were calculated using FMRIB’s Local Analysis of Mixed Effects (FLAME1). We also estimated voxel-wise correspondence between prosthesis usage and activity (versus baseline) in each of the three prosthesis conditions in one-handers, after partialling out voxel-wise activity in response to objects (versus baseline). As in the region of interest-based analysis, we only used one-handers eligible for functional MRI analysis (*n = *31). One-handers who brought their prosthesis to the study (*n = *25) were included in the ‘own’ prosthesis correlation analysis. Z-statistic images were minimally thresholded (Z > 2.3) and adjusted for multiple comparisons using whole-brain family-wise error (Gaussian Random Field) cluster size correction, and a corrected cluster significance threshold of *P < *0.05. For visualization purposes activation maps were projected onto an inflated cortex using FreeSurfer (Dale *et al.*, 1999; Reuter *et al.*, 2012) and Workbench (Van Essen *et al.*, 2013).

## Results

We first searched for group differences in visual hand-selective regions when participants were shown images of other people’s prosthesis images (active or cosmetic, Fig. 2A and B). Across all participants, unfamiliar active prosthesis images activated lateral occipito-temporal cortex, overlapping with hand-selective activity (Fig. 2C and D). Importantly, activity in the visual hand-selective regions of interest (Fig. 1A) was greater in one-handers, and prosthesis users in particular, compared to controls. A 2 × 2 ANOVA with factors group (all one-handers, *n = *31, controls, *n = *24) and prosthesis type (cosmetic, active) revealed a significant group difference [*F*(1,106) = 6.5, *P = *0.012], with one-handers showing stronger activity than controls. Although activity tended to be stronger for the cosmetic prostheses compared to active prostheses [*F*(1,106) = 3.5, *P = *0.066], the interaction term was not significant [*F*(1,106) = 0.4, *P = *0.538], indicating that this trend is likely driven by the visual features of the cosmetic prosthesis, which strongly resembles a hand. When focusing the analysis specifically on one-handers who are prosthesis users (*n = *26, Supplementary Table 2), we found significantly increased activity for images of unfamiliar active prostheses (who share little visual similarity with natural hands) in the hand-selective visual region of interest, compared with controls [*n = *24, *t*(48) = 2.8, *P = *0.008; Fig. 2E]. These results suggest that prosthesis usage leads to increased activity for prosthesis images in hand-selective regions.

We next determined whether prosthesis usage can account for interindividual differences in activity levels for prosthesis images in visual hand-selective regions of interest. Across one-handers, greater prosthesis usage in daily life positively correlated with increased activity in visual hand-selective cortex. This correlation was found when one-handers viewed images of both their own prosthesis [*n = *25, *r*(23) = 0.52, *P*_perm_ = 0.007, Fig. 3A–C], and another person’s active prosthesis, which shares the functionality of the hand but not its visual features [*n = *31, *r*(29) = 0.51, *P*_perm_ = 0.004, Fig. 3D–F]. Although usage showed only a trending correlation with activation to another’s cosmetic prostheses [*n = *31, *r*(29) = 0.33, *P*_perm_ = 0.068], correlations with active and cosmetic prosthesis images were not significantly different (two-tailed Fisher Z = 0.82, *P = *0.41). To examine the consistency of the effect across one-handers’ subgroups primarily using cosmetic or active prostheses, we repeated the analysis while accounting for prosthesis usage type (Table 1). We found that the relationship between prosthesis usage and visual activity remained significant [ANCOVA (*n = *26): images of active prostheses: *F*(1,23) = 7.1, *P = *0.014; images of cosmetic prostheses: *F*(1,23) = 6.0, *P = *0.022]. Importantly, the subgroup factor (prosthesis usage type) failed to show significance [*F*(1,23) = 0.13, *P = *0.72 and *F*(1,23) = 0.06, *P = *0.81, respectively]. As the effects were not restricted either to individual’s own prosthesis or to a prosthesis type primarily used by the one-hander (active versus cosmetic), these results hint at categorical changes in representation (van den Heiligenberg *et al.*, 2017), rather than familiarity with the specific visual features of the user’s prosthesis. This interpretation is consistent with the fact that one-handers routinely replace their prosthesis, and often use multiple prosthesis types (Table 1).

To determine whether the increased activity in prosthesis users could be ascribed to passive visual experience alone, we repeated the analysis in the control participants, based on their passive observation log scores (Supplementary Table 1). Although prosthesis-related experience is likely to be less intensive in controls compared to one-handers, the difference between the overall distribution of the POL and MAL scores was non-significant, as indicated by the Kolmogorov-Smirnov test (Z = 0.750, *P* = 0.627). Therefore, the correlation analysis is potentially suitable for measuring interindividual differences that scale with passive observation. Correlation found between prosthesis activity and controls’ visual experience was significantly smaller than the reported correlation between prosthesis activity and prosthesis usage in one-handers [*n = *21, active prosthesis: *r*(19) = 0.03, *P*_perm_ = 0.93, group difference: one-tailed Fisher Z = 1.76, *P = *0.039, Fig. 3G; cosmetic prosthesis: *r*(19) = −0.10; *P*_perm_ = 0.68, group difference: one-tailed Fisher Z = 2.13, *P = *0.017]. This result indicates that activity in hand-selective visual areas in the adult visual system scales with active everyday visuomotor experience.

To assess intrinsic coupling between individuals’ sensorimotor missing-hand area and bilateral hand-selective visual areas, we measured resting state functional connectivity. Visuomotor connectivity was correlated with prosthesis usage, while accounting for visuomotor connectivity with the intact hand area (see ‘Materials and methods’ section). One-handers who use their prosthesis more in daily life showed stronger relative connectivity than those with lower usage [*n = *31, *r*(29) = 0.55; *P*_perm_ = 0.001; Fig. 1B and C]. Here again, no significant correlation was found with controls’ visual experience [*n = *21, r(19) = −0.28, *P*_perm_ = 0.22, group difference: one-tailed Fisher Z = 3.0 *P = *0.001; Fig. 1D]. This result suggests that successful prosthesis usage is associated with increased visuomotor communication with the missing-hand’s territory.

To determine the utility of visuomotor functional connectivity in explaining variance in visual activity, a hierarchical regression analysis was implemented (see ‘Materials and methods’ section). Visual activity to (unfamiliar) active prostheses served as the dependent variable. As the sole predictor of such activity, usage accounted for 26% of the variance in the dependent variable [*n = *31, *F*(1,29) = 10.02, *P = *0.004], while visuomotor connectivity alone accounted for 22% of such variance [*F*(1,29) = 8.15, *P = *0.008]. When the predictors were combined, the explained variance was increased to 31% [*F*(2,28) = 6.24, *P = *0.006]. Considering this combined variance, we determined the shared versus unique effects of the predictors, i.e. the contribution of usage to activity that is unrelated to connectivity and vice versa. We found that the shared effect accounts for 17% of the variance in the activity. Connectivity uniquely accounted for only 5% of such variance (i.e. above the variance explained by usage), and this effect was not significant [*F*(1,28) = 2.1, *P = *0.161]. Importantly, the prosthesis usage also contributed little additional variance in activity (9%), beyond that explained by the connectivity. As above, the effect failed to reach significance level [*F*(1,28) = 3.6, *P = *0.068]. Although this analysis does not enable causal inferences to be drawn, it nevertheless clearly indicates a threefold coupling between activity, connectivity and usage.

Finally, we determined whether the repurposing of visual hand-selective areas to support prosthesis representation depends on the developmental period during which individuals experienced hand loss. We therefore repeated our main analysis showing correspondence between prosthesis daily usage and visual activity/connectivity (as described above), while accounting for any potential group differences between individuals with congenital and acquired handlessness. We found that the correspondence reported above remained significant, both with respect to activity [ANCOVA: own prosthesis (*n = *25): *F*(1,22) = 6.2, *P = *0.021; active prosthesis (*n = *31): *F*(1,28) = 9.0, *P = *0.006] and functional connectivity (*n = *31): [*F*(1,28) = 10.3, *P = *0.003]. Importantly, the subgroup factor (congenital versus acquired one-handedness) failed to show significance [own prosthesis: *F*(1,22) = 0.25, *P = *0.623; active prosthesis: *F*(1,28) = 0.06, *P = *0.804; functional connectivity: *F*(1,28) = 0.02, *P = *0.891]. This result is consistent with our previous findings showing that brain organization and reorganization in one-handers is best characterized by everyday experience (Makin *et al.*, 2013*a*; Hahamy *et al.*, 2015, 2017).

## Discussion

Here we show that prosthetic limbs, used to substitute the missing hand, can recruit brain resources normally devoted for body representation. We also show that this neurophysiological ‘embodiment’ of artificial limbs depends on prosthesis usage in everyday life—those individuals who rely more on their prosthesis to substitute hand function show stronger activity in hand-selective visual areas when presented with images of a prosthesis. Importantly, the engagement of hand-selective areas in prosthesis representation was not specific to individuals’ own prostheses, but generalized to other, unfamiliar, exemplars of artificial limbs. Furthermore, prosthetic limb representation did not depend on the type of prosthesis primarily used by each one-hander, or on the developmental period in which individuals lost their limb and started using prosthesis (i.e. congenital amelia versus acquired amputation in adulthood). Our findings therefore hint at categorical representation of artificial limbs that primarily depends on everyday experience (see van den Heiligenberg *et al.*, 2017 for related behavioural results). Finally, we show that prosthesis usage also shapes large-scale brain reorganization, specifically intrinsic connectivity between visual and sensorimotor hand-selective areas. Together, our findings provide the first account of how artificial limbs are represented in the brain of amputees.

While it has long been established that the lateral occipito-temporal cortex shows modular visual representation for upper limbs, the inputs guiding this representation, as well as its behavioural relevance, have been debated (Astafiev *et al.*, 2004; Peelen and Downing, 2005; Orlov *et al.*, 2010, 2014; Downing and Peelen, 2011; Bracci *et al.*, 2012; Gallivan and Culham, 2015; Lingnau and Downing, 2015; Zimmermann *et al.*, 2018). Indeed, recent studies have argued that visual body representation is independent of any motor experience (Vannuscorps and Caramazza, 2016; Striem-Amit *et al.*, 2017). We demonstrate that altered motor behaviour in daily life facilitates visual processing and shapes communication between visual and sensorimotor areas. However, since prosthesis usage strongly relies on visual input (due to lack of somatosensory feedback), people who use their prosthesis more in daily life also spend more time and attention while looking at it. It is therefore difficult to tease apart the contributions of pure visual experience as opposed to visuomotor experience. Several pieces of evidence in the current study can inform us on the potential contribution of active, versus passive, everyday experience. First, we found similar evidence when presenting individuals with images of their own prosthesis versus an unfamiliar one. This indicates that the increased activity doesn’t depend on visual familiarity *per se*. Second, we tested control participants with varying daily exposure to prosthesis usage, including family members and friends of one-handers. Even if these control participants may spend less time observing a prosthesis compared with prosthesis users, the lack of correlation between the degree of passive visual experience and activity in visual hand-selective areas for prosthesis images indicates that the two are not tightly coupled. Finally, our hierarchical regression analysis reveals that the re-appropriation of the visual hand-selective areas to support prosthetic limb representation depends not just on visual exposure to the prosthesis, but rather also on increased connectivity with hand-selective sensorimotor resources, induced by usage. While directionality cannot be inferred from these hierarchical analyses, our findings provide evidence for a tight coupling between daily actions, functional connectivity with sensorimotor cortex, and visual body representation.

In summary, our findings show that neurocognitive resources devoted to representing our body can support representation of artificial body parts. By providing first evidence for a relationship between embodied technology and successful prosthesis usage, our results may aid assistive and augmentative technological development and usage (Makin *et al.*, 2017).

## Funding

This work was supported by a Sir Henry Dale Fellowship jointly funded by the Wellcome Trust and the Royal Society (104128/Z/14/Z), an ERC Starting Grant (715022 EmbodiedTech), and a Cogito Foundation grant, awarded to T.R.M. S.N.M. was funded by a Natural Sciences and Engineering Research Council of Canada CREATE training program (371161-2009 CREAT) to J.C.C. H.J.B. is funded by a Wellcome Principal Research Fellowship (110027/Z/15/Z).

## Supplementary material

Supplementary material is available at *Brain* online.

## Supplementary Material

Supplementary Data

## Abbreviation

MAL: Motor Activity Log
