# Supplementary material for: Artificial limb representation in amputees
Source: Brain. 2018 Mar 9;141(5):1422–33. doi: 10.1093/brain/awy054 (PMC5917779; doi:10.1093/brain/awy054)
Supplement: Supplementary Data [file awy054_makin_2017_brain_supp_materials_submitted.pdf]

Supplementary materials: Artificial limb representation in amputees

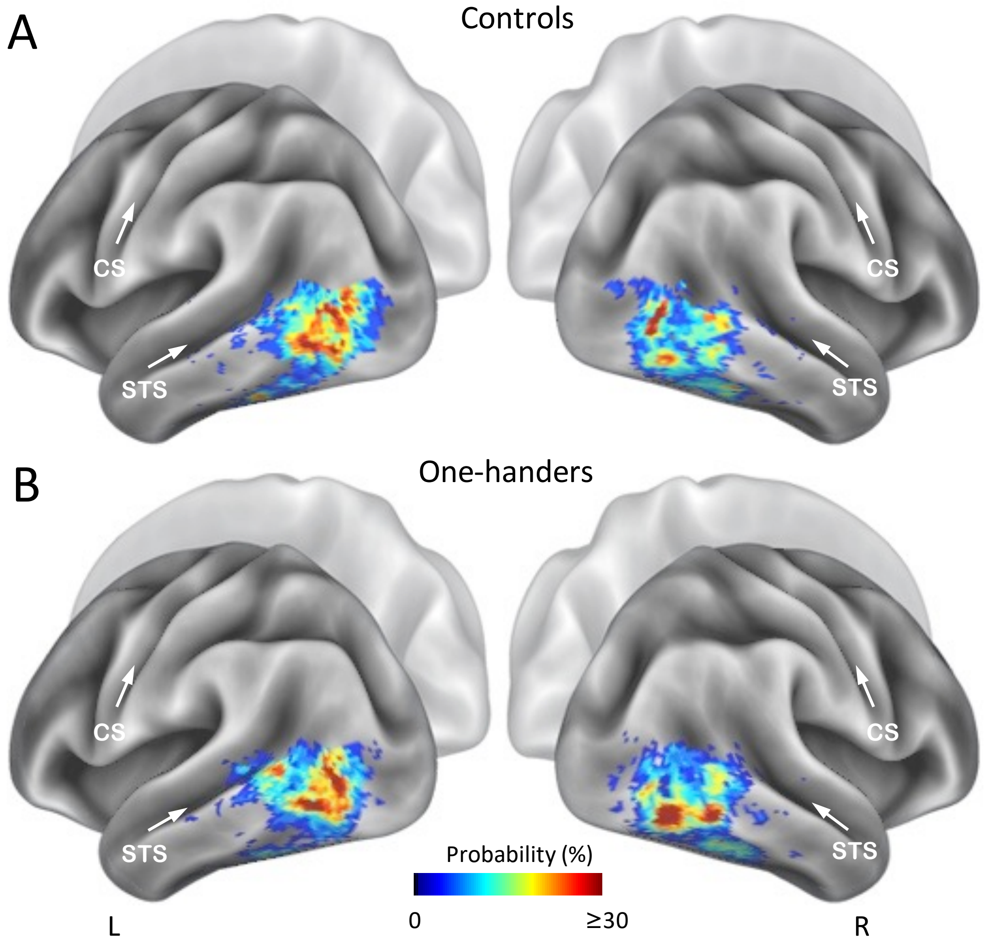

Supplementary Figure S1. Group probability maps for visual hand-selective regions of interest (ROIs) in control participants (A) and one-handers (B). All individual visual ROIs were superimposed per group, yielding corresponding probability maps. Warmer colours represent voxels that were included in greater numbers of individual ROIs.

Supplementary Table S1. Control participants Prosthesis Observation Log (POL) scores (equivalent to the MAL scores in **Table 1**) and prosthesis observation time in controls (equivalent to usage time in **Table 1**). POL: How frequently these participants observed a person use a prosthesis: 0-1: the scale of the observation frequency (using the same inventory of 27 daily activities as in **Table 1**). Observation time: 1-5: the scale for observation time of seeing a person use a prosthesis: 0=never; 1=rarely; 2=occasionally; 3=daily, less than 4 hours; 4=daily, between 4-8 hours, 5=daily, over 8 hours.

Subject	*Gender*	*Age*	*NonDominant Hand Side*	*Observation Frequency (POL)*	*Observation Time*
PC01	F	70	Left	0.15	2
PC02	F	49	Left	0.56	4
PC03	M	27	Left	0.17	1
PC04	F	36	Left	0.09	2
PC05	F	24	Left	0.13	2
PC06	M	25	Right	0	1
PC07	F	52	Left	0	0
PC08	F	43	Left	0	0
PC10	F	38	Left	0	1
PC11	M	52	Right	0	0
PC12	M	47	Left	0.09	1
PC13	M	41	Right	0	1
PC14	M	49	Left	0.02	1
PC15	M	28	Right	0	0
PC16	F	32	Left	0.31	4
PC17	F	42	Right	0.02	2
PC19	M	44	Right	0	0
PC20	F	40	Left	0	0
PC21	M	52	Right	0	0
PC22	F	62	Left	0.72	4
PC24	M	64	Right	0.24	5
PC09*	M	27	Left		
PC18*	M	31	Left		
PC23*	F	25	Left		

*These participants did not complete the prosthesis observation questionnaires.

Supplementary Table S2. Additional information about prosthesis usage and availability in one-handers (Note that neuroimaging data of 31 one-hander was included in the final fMRI analysis). 1 indicates inclusion in the specific class. Subj.=Subject, Pros.=Prosthesis, Cosm.=Cosmetic, Mech.=Mechanic, Myo.=Myoelectric, &=and, Cond.=Condition.

Subj.	*Pros. owner*	*Owner but not user*	*Pros. user: any pros.*	*Cosm. pros*	*Active prosthesis*				*Cosm. only*	*Active only*	*Both active & cosm.*	*Pros. in "own" cond.*
					Mech.	*Myo.*	*Both mech. &mio.*	*Any active pros.*				
PA01	1	0	1	1	0	0	0	0	1	0	0	1
PA02	1	0	1	1	0	0	0	0	1	0	0	1
PA03	0	0	0	0	0	0	0	0	0	0	0	0
PA04	1	0	1	1	1	0	0	1	0	0	1	1
PA06	1	0	1	1	1	0	0	1	0	0	1	1
PA07	1	0	1	0	1	0	0	1	0	1	0	0
PA08	1	0	1	1	1	0	0	1	0	0	1	1
PA09	1	0	1	1	1	0	0	1	0	0	1	0
PA10	1	1	0	0	0	0	0	0	0	0	0	1
PA11	1	0	1	1	1	0	0	1	0	0	1	1
PA12	1	0	1	0	1	0	0	1	0	1	0	0
PA13	1	1	0	0	0	0	0	0	0	0	0	1
PA14	0	0	0	0	0	0	0	0	0	0	0	0
PA15	1	0	1	0	1	1	1	1	0	1	0	1
PA16	1	0	1	1	0	0	0	0	1	0	0	1
PA17	1	1	0	0	0	0	0	0	0	0	0	1
PA18	1	0	1	1	0	0	0	0	1	0	0	1
PA19	1	0	1	1	0	0	0	0	1	0	0	1
PA20	1	0	1	1	0	0	0	0	1	0	0	1
PA21	1	0	1	1	0	0	0	0	1	0	0	1
PA22	1	0	1	1	0	0	0	0	1	0	0	1
PA23	1	0	1	0	1	0	0	1	0	1	0	1
PA24	1	0	1	0	0	1	0	1	0	1	0	1
PA25	1	0	1	0	0	1	0	1	0	1	0	1
PA27	1	0	1	1	0	1	0	1	0	0	1	1
PA28	1	0	1	0	0	1	0	1	0	1	0	1
PA29	1	0	1	0	1	0	0	1	0	1	0	0
PA30	1	0	1	0	1	1	1	1	0	1	0	1
PA31	1	0	1	1	0	0	0	0	1	0	0	1
PA32	1	0	1	1	0	0	0	0	1	0	0	1
PA33	1	0	1	0	1	0	0	1	0	1	0	1
Total (fMRI data)	**29**	**3**	**26**	**16**	**12**	**6**	**2**	**16**	**10**	**10**	**6**	**25**
PA05*	1	0	1	1	1	0	0	1	0	0	1	1
Total	**30**	**3**	**27**	**17**	**13**	**6**	**2**	**17**	**10**	**10**	**7**	**26**

*This participant was excluded from the fMRI analysis
